# Biomineralization of Glucose Oxidase from *Aspergillus niger* in ZIF-zni for Enhanced Biocatalytic Performance

**DOI:** 10.3390/bioengineering13040465

**Published:** 2026-04-16

**Authors:** Marija Stanišić, Milica Crnoglavac Popović, Nikola Knežević, Marko Radenković, Branimir Bajac, Olivera Prodanović, Radivoje Prodanović

**Affiliations:** 1 University of Belgrade-Faculty of Chemistry, Studentski trg 12, 11000 Belgrade, Serbia; mstanisic@chem.bg.ac.rs (M.S.); milicac@chem.bg.ac.rs (M.C.P.); 2 BioSense Institute, University of Novi Sad, Dr. Zorana Đinđića 1, 21000 Novi Sad, Serbia; nknezevic@biosense.rs (N.K.); marko.radenkovic@biosense.rs (M.R.); branimir.bajac@biosense.rs (B.B.); 3 University of Belgrade-Institute of Multidisciplinary Research, Kneza Višeslava 1, 11030 Belgrade, Serbia; oliverap@imsi.bg.ac.rs

**Keywords:** glucose oxidase, biomimetic mineralization, enzyme immobilization, ZIF-zni, metal–organic frameworks, enzyme@MOF biocomposites, heterogeneous biocatalysis

## Abstract

Biomineralization has recently emerged as a highly effective strategy for enzyme immobilization. Zeolitic imidazolate frameworks (ZIFs), a subclass of metal–organic frameworks (MOFs), are particularly attractive carriers due to their structural tunability and chemical stability. While ZIF-8 has been extensively studied, its denser and thermodynamically more stable analog ZIF-zni has received far less attention. In this work, we report the biomineralization of glucose oxidase (GOx) from *Aspergillus niger* within the ZIF-zni framework and systematically investigate the influence of zinc and imidazole (Im) concentration on immobilization performance. The optimized biocomposite, obtained at 10 mM Zn^2+^ and a Zn:Im ratio of 1:10, exhibited a specific activity of 2051 IU g^−1^, which is more than twice the activity obtained for GOx@ZIF-8 in our previous study (874 IU g^−1^). Furthermore, the GOx@ZIF-zni biocomposite demonstrated remarkable resistance to sodium dodecyl sulfate (SDS) and retained up to 50% of its activity after incubation at 65 °C for one hour. These results demonstrate that ZIF-zni is a highly promising carrier for enzyme immobilization and suggest that framework topology and synthesis conditions play a crucial role in determining the catalytic performance and stability of enzyme@MOF biocomposites.

## 1. Introduction

Because enzymes can catalyze complex chemical reactions under very mild reaction conditions, they are often referred to as biocatalysts from Mother Nature. Enzymes are characterized by high selectivity, activity, and specificity. Due to these characteristics, they have found applications in various fields, including food chemistry, medical chemistry, fine chemistry, the fuel industry, and environmental contamination control [1]. One essential flavoprotein enzyme of the glucose-methanol-choline (GMC) oxidoreductase family is glucose oxidase (EC 1.1.3.4, GOx). The source of GOx varies widely, including *Aspergillus niger*, *Aspergillus tubingensis*, and the genus *Penicillium*. However, glucose oxidase isolated from *Aspergillus niger* and *Penicillium nigricans* is the most widely used [2,3]. Glucose oxidase consists of two uniform subunits bound by hydrogen bonding and hydrophobic interactions, with each subunit containing a flavin adenine dinucleotide (FAD) and substrate-binding domain. This enzyme catalyzes the oxidation of β-D-glucose to D-gluconolactone, releasing H_2_O_2_ in a two-step reaction that utilizes molecular oxygen as an electron acceptor [4,5]. Since this enzyme is widely used in various industrial processes, its immobilization is a good strategy to protect it from harsh industrial conditions, such as organic solvents, high temperatures, and non-optimal pH.

Although the use of enzymes in industry has several advantages, free enzymes remain challenging to handle due to the potential for denaturation and loss of activity. Such problems can be overcome by immobilizing enzymes onto/into solid supports. Generally, there are three categories for immobilizing an enzyme, namely binding to a carrier, encapsulation, and cross-linking [6]. For the immobilization of an enzyme onto a solid support, several natural materials, such as collagen, alginate, and cellulose, as well as inorganic materials like zeolites and silica, can be used [7]. Through adsorption, covalent binding, pore entrapment, encapsulation, crosslinking, or ligand affinity, enzymes are immobilized within these carriers. Numerous methods can be employed in these processes, including electrospinning, 3D and laser printing, soft plasma polymerization, the use of crosslinked enzyme aggregates (CLEAs), nanoflowers, and metal–organic frameworks (MOFs) to create and prepare materials that harbor enzymes [8]. Generally, there are two dominant strategies for encapsulating an enzyme. The first method involves direct inclusion or spontaneous diffusion of the enzyme into the pre-synthesized porous structure. This method is most often used for immobilizing enzymes into mesoporous (alumino) silica, cationic clay minerals, and, in recent years, metal–organic frameworks and covalent organic frameworks (COFs) [9,10]. For this method of immobilization, it is necessary to match the size of the pores in the support with that of the enzyme, ensuring the diffusion of the substrate to the active site [11,12]. The second method represents in situ encapsulation, in which the immobilization procedure and matrix synthesis are performed simultaneously. Although this approach circumvents the size limitation problem, it still presents a fundamental synthetic challenge, as the host matrix synthesis must occur under acceptable conditions (such as aqueous solutions, low pH, and low temperature) to prevent enzyme denaturation [13]. For this method of immobilization, MOFs and hydrogen-bonded organic frameworks (HOFs) are most commonly used due to their porous structures.

MOFs are fabricated hybrid crystalline structures composed of metal ions and organic linkers, produced through coordination chemistry. They have developed into an important class of porous materials with a range of micro- and nanostructures and versatile chemistry since their discovery in the 1980s [14]. Various MOF types have been produced using organic ligands and numerous metal ions, including iron, copper, zinc, and rare-earth ions [15]. Additionally, MOFs possess several advantages, including a large specific surface area and ultra-high porosity. However, several disadvantages exist, including poor chemical and hydrothermal stability, limited mechanical strength, and limited use [16,17,18]. To achieve greater catalytic activity, MOFs can control the enzyme’s conformation. The enzymes’ conformational changes and their activity are reduced or even lost when exposed to harsh conditions, such as high pH, high temperatures, or organic solvents [19]. Research indicates that ZIF-based biomimetic mineralization is the most favorable and effective method of MOF synthesis compared to other techniques, such as surface conjugation and pore immobilization [20,21]. Although ZIF-8 is the best described and researched so far, there are many more ZIFs, such as ZIF-zni, ZIF-7, ZIF-20, ZIF-72, ZIF-77, and ZIF-90. Because of the ability to nucleate proteins in aqueous solution and to form enzyme@ZIF structures, Huang et al. examined various properties of the resulting biocomposites. Bioactivity, protein encapsulation effectiveness, and mineral crystallinity and defect degree are analyzed. Additionally, using a variety of spectroscopic techniques, the conformations of proteins released from or encapsulated in three selected ZIFs are carefully studied. These experiments demonstrated that the stability of the enzyme within the biocomposite is determined by the size, dimensions, and chemical microenvironment of the ZIF pores [22].

In this work, GOx@ZIF-zni biocomposites were prepared and systematically characterized to investigate the influence of synthesis conditions on immobilization behavior and catalytic performance. ZIF-zni, composed of zinc ions and imidazolate ligands, represents the thermodynamically most stable and densest ZIF topology and is generally considered non-porous. The effects of synthesis conditions on enzyme incorporation, activity, and stability were evaluated, and the performance of GOx@ZIF-zni was compared with previously reported GOx@ZIF-8 systems. The results demonstrate that, despite its dense structure, ZIF-zni can provide enhanced specific activity and improved stability of the immobilized enzyme.

## 2. Materials and Methods

### 2.1. Materials

Glucose oxidase from *Aspergillus niger* (GOx), peroxidase from horseradish (HRP), and imidazole (Im) were obtained from Sigma-Aldrich (St. Louis, MO, USA), while 2, 2′-azino-bis (3-ethylbenzothiazoline-6-sulfonic acid) (ABTS) was purchased from AppliChem (Darmstadt, Germany). Sodium metaperiodate (NaIO_4_) was purchased from VWR Chemicals (Leuven, Belgium). D-glucose monohydrate and zinc acetate dihydrate (Zn(O_2_CCH_3_)_2_·2H_2_O) were obtained from Lachner (Neratovice, Czech Republic). Sodium dodecyl sulfate (SDS) was obtained from Serva (Heilderberg, Germany). All reagents were analytical grade, and all solutions were prepared with distilled water.

### 2.2. Biomimetic Mineralization

The stock solutions of native glucose oxidase and Zn(OOCCH_3_)_2_·2H_2_O were mixed for one minute. After one minute of stirring, the reaction mixture was added to the imidazole solution and mixed for 30 min. In this procedure, the final concentration of the enzyme was 0.35 mg/mL. The final concentrations of zinc acetate were 10, 20, and 60 mM. For imidazole solutions, the final concentrations were 10, 20, 50, and 100 times higher than those of the zinc acetate solution. After stirring, the suspensions were left at room temperature for 12 h. Thereafter, the reaction mixtures were centrifuged at 6000 rpm for 10 min. Afterward, the supernatants were collected, and all precipitates were washed three times with distilled water. The washed precipitates were incubated in a 10% *w*/*v* SDS solution for 30 min. After that, suspensions were centrifuged, supernatants were rejected, and precipitates were washed two times with 0.1 M sodium acetate buffer (pH = 5.5), two times with 96% *w*/*v* ethanol, and one time with distilled water.

### 2.3. Material Microstructure and Crystal Structure Analysis

Particle morphology and size were analyzed by scanning electron microscopy (SEM) using a ThermoFisher Scientific Apreo 2C, Waltham, MA, USA at 10,000× magnification and 1 kV. No sample conductive coating was applied before measurements. The study of the materials’ crystal structures was performed using a Rigaku Smartlab, Tokyo, Japan with Bragg–Brentano geometry for powder samples in 1D detector mode. Samples were recorded in continuous mode at 5 °C/min over a 2θ range of 5–50°.

### 2.4. BET Analysis

Nitrogen physisorption measurements were performed on NOVAtouch LX2 (Anton Paar, Graz, Austria) at 77 K. Before the gas sorption experiment, the material was dried and degassed under vacuum at 130 °C for 3 h. The Brunauer–Emmett–Teller (BET) surface areas were calculated from the nitrogen adsorption isotherms in the P/P0 range from 0.015 to 0.07. Total pore volumes were calculated from the nitrogen sorption isotherms at P/P0 = 0.95.

### 2.5. Activity Measurements

The activity was measured with ABTS as a substrate, as previously described [23]. The reaction mixture for activity measurements contained 1 mM ABTS, 1 U/mL HRP, and 0.25 M D-glucose in 0.1 M sodium acetate buffer (pH = 5.5). The activity was determined by monitoring a change in absorbance at 405 nm, using an extinction coefficient of 36.8 mM^−1^ cm^−1^ for oxidized ABTS. In these measurements, 1 U of activity is defined as the amount of enzyme that converts 1 µmol of D-glucose per min at 25 °C.

The immobilization performance parameters (YA, YP, Ploading, specific activity, and effectiveness factor, η) were calculated as described in Section 3.2 and based on previously reported methods.

### 2.6. pH Activity and Stability Measurements

The activity was measured with ABTS as a substrate. The reaction mixture for pH activity measurements contained 1 mM ABTS, 1 U/mL HRP, and 0.25 M D-glucose in 0.05 M sodium acetate–phosphate–glycinate buffer (pH = 3.5–8.5). For stability assessment, the biocomposite was incubated in a buffer for 30 min, and activity was measured in 0.1 M sodium acetate buffer (pH 5.5) as previously described.

### 2.7. Thermal Stability Measurements

Thermostability of all samples was determined by incubation in water at 65 °C for 1 h, and specific activity was measured as previously described. The residual activity of samples was defined as the ratio of specific activities before and after incubation at 65 °C.

## 3. Results

Previous research, including ours, has demonstrated that immobilizing glucose oxidase in ZIF-8 provides mild immobilization with enhanced stability against temperature and detergents [23]. However, the literature reports that some enzymes, such as laccase, can be immobilized in other ZIFs with topologies and porosities different from the *sod* topology of ZIF-8. There are reports that laccase could be immobilized using biomineralization with Zn^2+^ and imidazole (Im) in ZIFs with zni topology [24]. Several synthetic methods for ZIF-zni have been developed since its initial announcement in 1980 [25]. Additionally, Liang et al. demonstrated that the ratio of zinc and 2-methylimidazole may influence the distribution and structure of biomacromolecules in protein ZIF-8 biocomposites [26]. Consequently, to optimize the immobilization of GOx, we conducted experiments to investigate the effect of changing the ligand used in biomineralization and varying the Zn:Im ratio on the structure and activity of the biocomposites. The final concentration of glucose oxidase in all experiments was 0.35 mg/mL. Also, the final concentrations of zinc(II) ions were 10, 20, and 60 mM. The Zn:Im ratios were 10, 20, 50, and 100. As in the previous experiments, the aqueous zinc solution was mixed with the protein solution, and the mixture was then added to imidazole. The order of component addition results from zinc ions interacting with the protein surface via electrostatic interactions. Then, after adding the imidazole solution, a coordinate covalent bond forms between the imidazole and the zinc.

### 3.1. Structural Characterization

**Figure 1:**
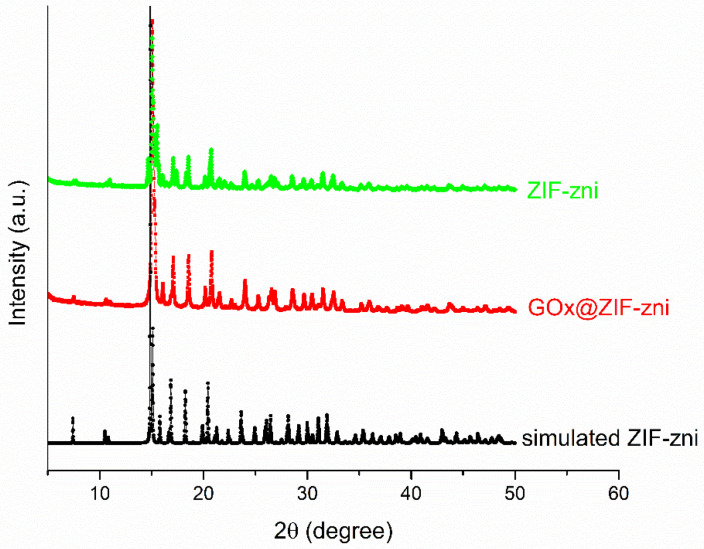
Simulated PXRD of the simulated ZIF-zni, ZIF-zni, and the GOx@ZIF-zni biocomposite obtained with 20 mM Zn^2+^ and a Zn:Im ratio of 1:50, washed with distilled water, and air dried.

After synthesis, the biocomposites were washed with distilled water and characterized using powder X-ray diffraction (PXRD), SEM, and the Brunauer–Emmett–Teller (BET) method to determine their topology. PXRD analysis confirmed that samples adopted the zni topology, as indicated by diffraction patterns consistent with those of simulated ZIF-zni (Figure 1).

Both experimental samples show the characteristic reflections of the zni topology with a major peak at approximately 15.2° (2θ), corresponding to the (110) plane, in good agreement with the simulated pattern obtained from the reported CIF structure of ZIF-zni [25]. The preservation of these reflections confirms the formation of the ZIF-zni phase and indicates that the enzyme encapsulation process did not alter the crystalline framework. Compared to ZIF-zni, the GOx@ZIF-zni biocomposite exhibits a slightly reduced peak intensity and minor broadening, consistent with a small decrease in crystallinity due to the incorporation of enzyme molecules into the framework during biomineralization.

**Figure 2:**
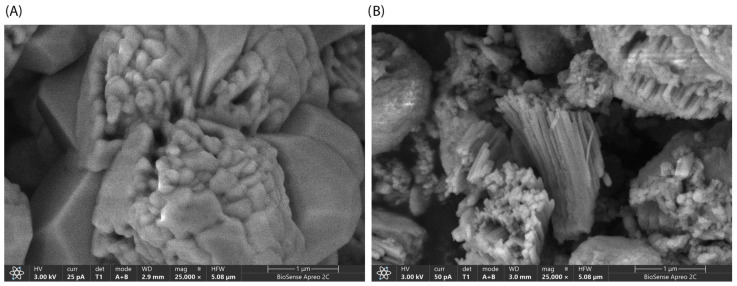
SEM images of ZIF-zni and GOx@ZIF-zni biocomposites obtained with 20 mM Zn^2+^ and a ratio Zn:Im of 1:50, washed with distilled water, and air dried: (**A**) ZIF-zni; (**B**) GOx@ZIF-zni.

SEM imaging revealed crystals with uniform, densely packed prismatic morphology, smooth surfaces, and sharp edges (Figure 2).

SEM analysis (Figure 2) revealed that both ZIF-zni and GOx@ZIF-zni samples possess prismatic crystal morphology characteristic of the zni topology. However, the GOx@ZIF-zni particles are slightly smaller and exhibit surface irregularities compared to ZIF-zni, suggesting that the incorporation of glucose oxidase during synthesis affects the crystal growth dynamics and leads to partial roughening of the crystal surfaces. These observations are consistent with the PXRD results, which also indicated a slight decrease in crystallinity after enzyme encapsulation.

**Figure 3:**
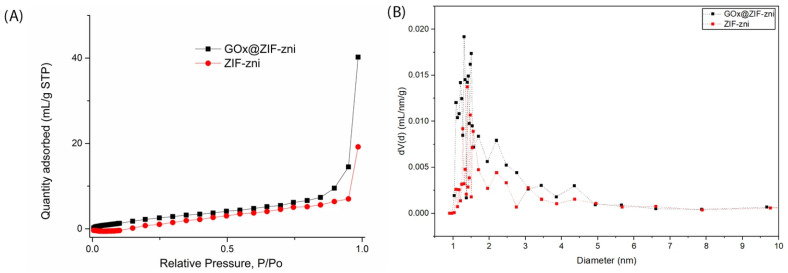
(**A**) N_2_ adsorption–desorption isotherms of ZIF-zni and GOx@ZIF-zni biocomposites recorded at 77 K. (**B**) BJH pore size distribution of ZIF-zni and GOx@ZIF-zni in the mesoporous range (1–10 nm). ZIF-zni and GOx@ZIF-zni biocomposites were obtained with 20 mM Zn^2+^ and a Zn:Im ratio of 1:50, washed with distilled water, and air-dried.

The N_2_ adsorption–desorption isotherms of ZIF-zni and GOx@ZIF-zni (77 K) show typical type I behavior, confirming the microporous nature of both materials (Figure 3A).

The BET surface area of GOx@ZIF-zni was determined to be 5.32 m^2^/g. In contrast, a physically meaningful BET fit could not be obtained for ZIF-zni because nitrogen adsorption was extremely low over the applicable relative pressure range, rendering the BET model non-physical. The observed surface area of GOx@ZIF-zni is attributed to enzyme-induced structural heterogeneity, including interparticle voids and defect-related surface contributions, rather than intrinsic framework porosity.

Although ZIF-zni is generally considered a dense and nearly non-porous framework, the GOx@ZIF-zni biocomposite exhibits a slightly higher total pore volume (0.0225 mL/g) compared to ZIF-zni (0.0109 mL/g). This increase can be attributed to structural modifications induced during biomimetic mineralization, where the presence of glucose oxidase influences nucleation and crystal growth, leading to the formation of interparticle voids and structural defects.

The BJH pore size distribution (Figure 3B) further supports this observation, indicating a more pronounced contribution of mesoporous features in GOx@ZIF-zni. While both samples retain predominantly microporous characteristics, the BJH analysis reveals additional textural mesoporosity arising from interparticle voids and defect-related structures. A clear difference in pore size distribution is observed between the two samples. ZIF-zni exhibits a broader and more dispersed distribution across a wider diameter range, whereas GOx@ZIF-zni shows a more pronounced contribution in the small pore diameter region (~1–2 nm). This suggests that enzyme incorporation not only blocks the pore system but also alters the crystallization pathway, promoting the formation of additional small textural pores and defect-related voids. These structural modifications may facilitate substrate diffusion via defect-mediated pathways, consistent with the biocomposite’s preserved catalytic activity after washing surface-adsorbed enzyme molecules.

The textural differences are consistent with SEM observations (Figure 2), which reveal that GOx@ZIF-zni contains a higher proportion of fractured and irregular prismatic particles compared to the well-defined crystals of pure ZIF-zni. Such morphological disruption indicates less compact packing and a more open microstructure, explaining the increased pore volume and improved textural accessibility despite enzyme incorporation.

Overall, the combined BET, BJH, and SEM analyses demonstrate that enzyme incorporation into the ZIF-zni framework alters crystal growth and morphology, resulting in a structurally heterogeneous material with enhanced substrate accessibility, which is favorable for biocatalytic applications.

### 3.2. Kinetic Immobilization Performance Parameters

Biocomposites’ enzyme activity and thermostability after washing with water and 10% (*w*/*v*) SDS were determined. Since GOx@ZIF-zni biocomposites represent heterogeneous structures that can be characterized by various parameters, a thorough assessment of the main characteristics of the immobilized enzyme is necessary [27]. As in our previous work, we used several key immobilization parameters to characterize biocomposites, as described in the literature. Activity balance (Y_A_) is defined as the ratio of immobilized enzyme activity to the enzyme activity used for immobilization ($\frac{Immobilized\;enzyme\;activity\;(U)}{Applied\;enzyme\;activity\;(U)}\times 100\%$). Protein balance (Y_P_) represents the ratio of the total immobilized amount of protein and the amount of protein used in the biomineralization process ($\frac{Mass\;of\;immobilized\;protein\;(g)}{Mass\;of\;used\;protein\;(g)}\times 100\%$) [28]. Protein loading (P_loading_) represents the mass of immobilized protein (enzyme) per mass of biocomposite and is expressed in mg/g ($\frac{Mass\;of\;immobilized\;protein\;in\;biocomposite\;(mg)}{Mass\;of\;biocomposite\;(g)}$) [29]. Specific activity of the biocomposite is defined as the amount of active enzyme per mass of solid biocomposite ($\frac{Enzyme\;activity\;of\;biocomposite\;(U)}{Mass\;of\;biocomposite\;(g)}$), while the specific activity of bound enzyme is defined as the activity of immobilized enzyme per mg immobilized enzyme ($\frac{Specific\;activity\;of\;biocomposite\;(U/g)}{Mass\;of\;immobilized\;enzyme\;in\;biocomposite\;(mg/g)}$) [27]. The effectiveness factor (ƞ) is the ratio of the specific activity of the bound enzyme and the specific activity of the free soluble enzyme ($\frac{Specific\;activity(\frac{U}{mg}enzyme\;bound)}{Specific\;activity(\frac{U}{mg}free\;enzyme)}$ × 100%) [30]. These parameters provide complementary information on immobilization efficiency, enzyme distribution, and catalytic performance, enabling a comprehensive evaluation of enzyme@MOF biocomposites [27].

For samples prepared at high imidazole concentrations, determination of protein concentration after biomineralization was unreliable due to imidazole interference; therefore, immobilization yield values for these samples are not reported. Consequently, immobilization efficiency in this study was evaluated primarily through catalytic activity and stability measurements, which are more relevant functional parameters for assessing the performance of enzyme@MOF biocomposites (Table 1, Table 2 and Table 3).

The data presented above show that the specific activity of the biocomposites increases as the zinc-to-imidazole ratio decreases at higher Zn^2+^ concentrations, where the corresponding imidazole concentrations are significantly higher. At lower Zn^2+^ concentrations, this trend is less pronounced, indicating that the effect depends on both the Zn:Im ratio and the absolute imidazole concentration. These results are most likely due to an excessively high concentration of imidazole in the reaction mixture. Namely, Huang et al. showed that elevated concentrations of imidazole and its derivatives in the reaction mixture led to enzyme denaturation due to protonation and/or the competitive coordination effect of these compounds [22]. As an obvious consequence of the influence of an excessively high imidazole concentration on enzyme denaturation, a biocomposite obtained using a final zinc concentration of 60 mM and a Zn:Im ratio of 100 had the smallest specific activity (Table 3).

**Figure 4:**
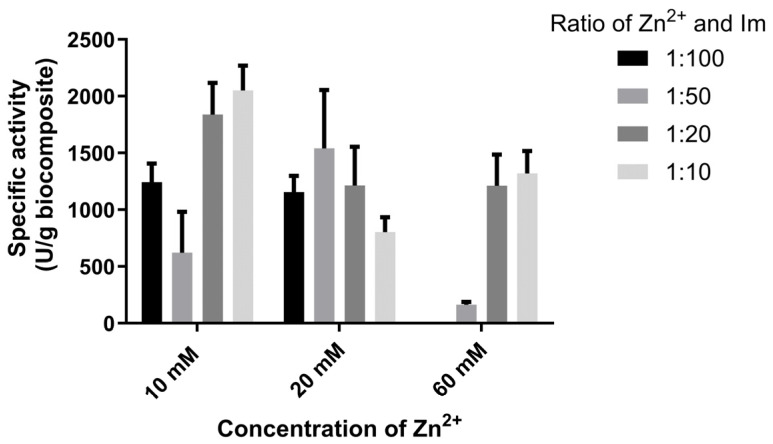
Specific activity of GOx@ZIF-zni biocomposites, after washing only with water.

However, as shown in Figure 4, the best results for the biocomposite’s specific activity are obtained when zinc and imidazole are used at a 1:10 ratio, yielding a final zinc concentration of 10 mM.

Unlike the specific activity of the biocomposites, the highest specific activity of the bound enzyme was observed with zinc and imidazole at a 1:10 ratio, and a final zinc concentration of 60 mM. A similar trend was observed for the effectiveness factor.

For the same conditions used in our previous work with 2-methylimidazole (20 mM Zn^2+^, 1:50 Zn:HmIM), we obtained with imidazole (GOx@ZIF-zni) twice the specific activity of the biocomposite washed only with water, 1540 IU/g vs 815 IU/g [23]. By further optimizing the zinc concentration (10 mM) and the zinc-to-imidazole ratio (1:10), we obtained a biocomposite with a higher specific activity of 2051 IU/g. Other kinetic parameters (Y_A,_ Y_P_, and P_loading_) were similar to our previous research [23].

**Figure 5:**
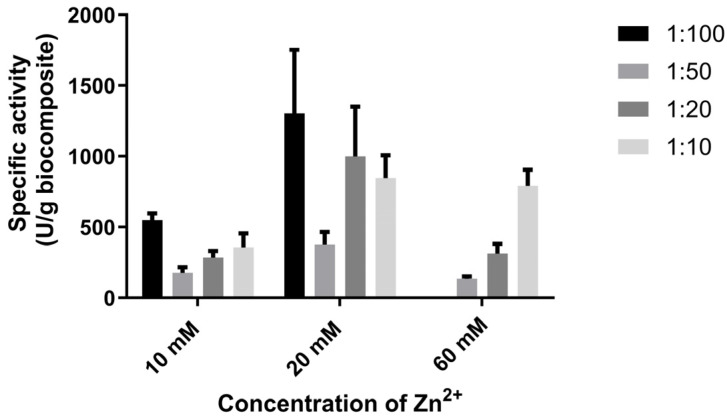
Specific activity of GOx@ZIFzni biocomposites after washing with distilled water and 10% SDS.

To eliminate the activity of biocomposites arising from surface-adsorbed enzyme, they were incubated in 10% (*w*)/*)v*) SDS for 30 min. After removing the SDS with distilled water, ethanol, and buffer, the specific activity was measured again using the previously described protocol. The data are presented in Figure 5. show that the best results are obtained at a final zinc concentration of 20 mM, at which most biocomposites retain a high specific activity even after washing with SDS, except when the imidazole concentration is 50 times higher than zinc.

The highest specific activity of GOx@ZIF-zni biocomposite (1305 U/g) after washing surface adsorbed enzyme with SDS was obtained using 20 mM Zn^2+^ and Zn:Im ratio of 1:100. Using the same conditions as in our previous work with ZIF-8 (20 mM zinc and Zn:HmIM 1:50 ratio) we obtained specific activity for GOx@ZIFzni of 376 U/g that was 24 times higher than the 16 U/g that we previously reported [23]. This could be due to the higher density of the zni topology, which better protects the enzyme from SDS washing and denaturation than the sod topology of ZIF-8. In some cases, the apparent increase in activity after SDS washing can be attributed to the removal of loosely bound or surface-adsorbed enzyme molecules and to improved accessibility of active sites. The relatively large standard deviations further reflect the structural heterogeneity of the biocomposites and variability in enzyme distribution, consistent with the heterogeneous nature of enzyme@MOF biocomposites.

### 3.3. pH Activity and Stability

**Figure 6:**
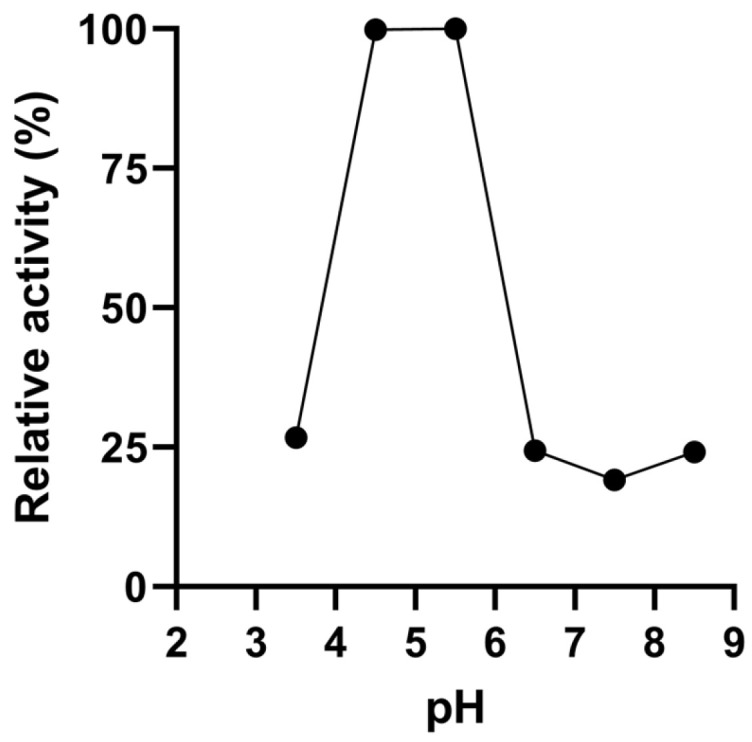
Dependence of GOx@ZIFzni biocomposite obtained with 20 mM Zn^2+^ and a Zn:Im ratio of 1:50, washed with distilled water, versus pH after washing only with distilled water.

The effect of pH from 3.5 to 8.5 on the catalytic activity of GOx@ZIF-zni biocomposites was evaluated by measuring activity at different pHs in 50 mM sodium acetate–phosphate–glycinate buffer (Figure 6).

The immobilized enzyme exhibited a well-defined optimum similar to that of the free enzyme between pH 4.5 and 5.5, indicating that biomimetic mineralization preserves the native catalytic properties of GOx [5].

When the biocomposite was incubated in the same buffer system over the pH range of 3.5–8.5, no detectable loss of activity (30 min incubation) or change in wet mass (6 h incubation) was observed, indicating high pH stability of the immobilized enzyme and preservation of the biocomposite structure, with no evidence of significant loss of its components under these conditions.

### 3.4. Thermal Stability of Biocomposites

**Figure 7:**
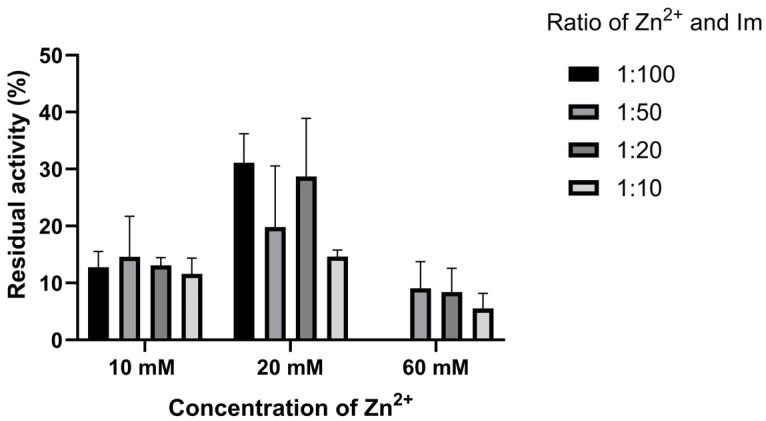
Residual activity of GOx@ZIF-zni biocomposites washed only with distilled water after incubation for one hour at 65 °C.

**Figure 8:**
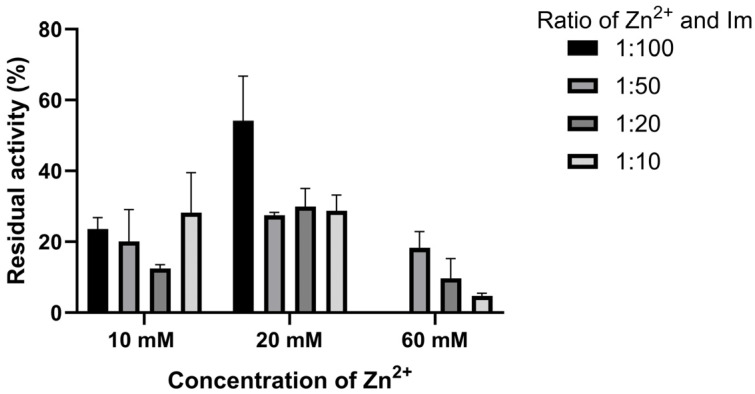
Residual activity of GOx@ZIF-zni biocomposites washed only with distilled water and 10% (*w*/*v*) SDS after incubation for one hour at 65 °C.

The thermostability of all biocomposites is presented as a residual activity, which represents the ratio of the biocomposites’ specific activity after incubation of the sample at 65 °C for 1 h to the particular activity of the non-incubated sample. Thermostability was measured for both biocomposites washed only with distilled water (Figure 7) and with 10% (*w*/*v*) SDS (Figure 8).

The results show that the samples’ thermostability is highest in the biocomposites, with a final zinc concentration of 20 mM. Also, reducing the imidazole concentration decreases thermostability. Since similar results were obtained for the biocomposite’s specific activity after SDS washing, it can be assumed that most enzyme molecules are immobilized on its surface (Figure 7 and Figure 8). The highest thermostability was observed in the biocomposite, with a final zinc concentration of 20 mM and an imidazole concentration 100 times higher. After incubation at 65°C for 1 h, this biocomposite retained about 30% of its initial specific activity.

After washing the biocomposites with SDS, higher thermostabilities were observed compared with those washed only with distilled water. Namely, the highest thermostability was observed in the biocomposites obtained using a final zinc concentration of 20 mM and an imidazole concentration 100 times higher. This biocomposite, after washing with SDS and incubation at 65 °C for 1 h, retained about 50% of its specific activity, compared with 30% for the biocomposite washed only with water. This trend was similar to our previous research and to the result of removing surface-adsorbed enzyme molecules, but the residual activity of 50% for GOx@ZIF-zni was higher than the 30% we reported for GOx@ZIF-8 washed with water and SDS [23].

In our previous work, further enhanced thermostability was achieved using periodate-oxidized GOx, which enabled additional cross-linking; however, in the present study, native GOx was used to specifically evaluate the effects of framework topology and synthesis conditions on enzyme stabilization.

These results are most likely due to better incorporation of enzymes into the interior of ZIF-zni, which further stabilized the enzyme against thermal denaturation compared to the *sod* topology of ZIF-8.

## 4. Discussion

The results obtained in this study demonstrate that both the composition of the synthesis mixture and the framework topology play a crucial role in determining the catalytic performance of enzyme@MOF biocomposites. In contrast to our previous work on GOx@ZIF-8, where the sodalite topology provided a relatively open and porous structure, the present study focuses on ZIF-zni, a denser and thermodynamically more stable framework.

Despite their nominally non-porous nature, the GOx@ZIF-zni biocomposites exhibited higher specific activity than GOx@ZIF-8. This apparent contradiction suggests that catalytic performance is not governed solely by intrinsic framework porosity but by a combination of structural defects, interparticle voids, and local microenvironmental effects arising during biomimetic mineralization. SEM and BET analyses support this interpretation, indicating the presence of structural irregularities and additional accessible volume in the biocomposite, which likely facilitate substrate diffusion. Therefore, GOx@ZIF-zni exhibited slightly higher N_2_ uptake than ZIF-zni without the enzyme. Given the intrinsically dense and non-porous nature of the zni topology, this increase cannot be attributed to the formation of larger intrinsic pores, but rather to defect formation and interparticle voids generated during biomimetic mineralization. This observation provides direct experimental support for the proposed diffusion pathways responsible for the observed catalytic activity. It should be noted, however, that these defect- and void-mediated pathways are inherently heterogeneous and difficult to quantify, and their contribution to mass transport may vary depending on synthesis conditions and enzyme distribution. Therefore, the observed catalytic activity cannot be attributed solely to intrinsic framework properties but rather to a combination of structural heterogeneity, defect formation, and local microenvironmental effects.

The observed dependence of activity on the Zn:Im ratio further supports the importance of the chemical environment during synthesis. At high imidazole concentrations, a decrease in activity was observed, which can be attributed to enzyme destabilization or partial denaturation, as previously reported in the literature. Lower imidazole concentrations and moderate Zn^2+^ levels (10 mM, Zn:Im = 1:10) provided optimal conditions for preserving enzyme activity while enabling efficient incorporation into the framework.

A key finding of this study is the significantly improved resistance of GOx@ZIF-zni biocomposites to SDS treatment. Since SDS removes surface-adsorbed proteins, the retained activity after SDS washing can be attributed primarily to encapsulated enzyme molecules. Higher residual activity compared to GOx@ZIF-8 suggests that the denser zni topology provides more effective physical entrapment and protection of the enzyme within the framework. This is consistent with previous findings showing that stronger enzyme–framework interactions and reduced pore accessibility can enhance enzyme stability under harsh conditions.

Thermal stability results further support this conclusion. The increased residual activity after incubation at elevated temperatures indicates that the ZIF-zni framework provides a more stabilizing microenvironment than ZIF-8. This effect can be attributed to the enzyme’s reduced conformational flexibility within the dense framework, as well as to protection from solvent-induced denaturation.

Taken together, these results show that a balance between accessibility and protection governs the performance of enzyme@MOF biocomposites. While more open frameworks, such as ZIF-8, may favor diffusion, denser frameworks, such as ZIF-zni, can provide superior enzyme stabilization and, under optimized synthesis conditions, even higher catalytic performance. This suggests that framework topology should be considered a key parameter in the rational design of enzyme@MOF biocatalysts. In particular, the present results demonstrate that even a dense, nominally non-porous ZIF topology can support efficient entrapment immobilization and enhanced biocatalytic activity when structural heterogeneity and defect-mediated accessibility are introduced during biomimetic mineralization.

Finally, this study opens new perspectives for the rational design of enzyme@MOF systems by combining control over framework topology with optimization of synthesis conditions. Future work should focus on elucidating the relationships among defect formation, enzyme distribution, and catalytic performance, and on extending this approach to other enzymes and ZIF topologies.

## 5. Conclusions

In this study, we demonstrated that both synthesis conditions and framework topology critically determine the performance of enzyme@MOF biocomposites. Optimization of the Zn^2+^ concentration and Zn:Im ratio enabled the preparation of GOx@ZIF-zni biocomposites with significantly enhanced catalytic activity (2051 IU/g) compared to previously reported GOx@ZIF-8 systems, especially after washing surface adsorbed enzyme molecules with SDS (376 U/g).

Although ZIF-zni is a dense and nominally non-porous framework, the resulting biocomposites exhibited superior catalytic performance and improved thermal and SDS stability. These effects can be attributed to enzyme-induced structural heterogeneity, including defect-related accessibility, interparticle voids, and favorable microenvironmental effects that promote substrate diffusion while enhancing enzyme entrapment and stabilization.

Overall, these results highlight ZIF-zni as a promising platform for enzyme immobilization and demonstrate that even dense MOF topologies can outperform more porous structures when synthesis conditions are properly optimized. The study further emphasizes framework topology as an important design parameter in the development of advanced enzyme@MOF biocatalysts.

## Figures and Tables

**Table 1:** Key immobilization performance parameters for biocomposites of GOx@ZIF-zni obtained with 10 mM Zn^2+^.

Parameter/Ratio of Zn:Im	1:100	1:50	1:20	1:10
Y_A_ (%)	91.51 ± 7.52	88.53 ± 11.05	85.79 ± 10.95	90.81 ± 4.79
Y_P_ (%)	88.85 ± 14.62	82.72 ± 15.06	90.31 ± 9.68	96.08 ± 3.67
P_loading_ (mg/g_carrier_)	223.30 ± 62.15	463.65 ± 14.71	210.91 ± 76.44	219.43 ± 25.70
Specific activity bicomposite washed dH_2_O (U/g_biocomposite_)	1243.19 ± 163.44	622.30 ± 360.11	1839.31 ± 277.49	2051.45 ± 218.79
Specific activity (U/mg_enzyme bound_)	4.92 ± 2.52	1.33 ± 0.73	9.69 ± 4.09	12.50 ± 6.53
η (%)	4.44 ± 2.08	minus 1.281.64 ± 1.28	9.04 ± 1.73	7.45 ± 1.58
Specific activity bicomposite washed dH_2_O and 10% SDS (U/g_biocomposite_)	548.63 ± 48.31	177.77 ± 39.68	85.87 ± 46.67	357.78 ± 97.28

**Table 2:** Key immobilization performance parameters for biocomposites of GOx@ZIF-zni obtained with 20 mM Zn^2+^.

Parameter/Ratio of Zn:Im	1:100	1:50	1:20	1:10
Y_A_ (%)	99.37 ± 0.55	99.07 ± 1.30	92.99 ± 6.85	79.58 ± 13.05
Y_P_ (%)	n.d.	95.15 ± 4.04	98.61 ± 2.41	96.32 ± 3.17
P_loading_ (mg/g_carrier_)	n.d.	189.30 ± 19.53	157.57 ± 34.69	125.06 ± 9.28
Specific activity bicomposite washed dH_2_O (U/g_biocomposite_)	1155.93 ± 142.79	1539.75 ± 514.97	1214.01 ± 341.89	802.92 ± 131.89
Specific activity (U/mg_enzyme bound_)	n.d.	8.14 ± 2.55	7.79 ± 1.76	9.58 ± 5.44
η (%)	n.d.	8.84 ± 1.94	8.38 ± 2.03	9.85 ± 4.00
Specific activity bicomposite washed dH_2_O and 10% SDS (U/g_biocomposite_)	1304.88 ± 447.30	375.96 ± 89.23	998.60 ± 354.05	848.05 ± 160.03

n.d., not determined due to interference from imidazole in protein concentration measurements after biomineralization.

**Table 3:** Key immobilization performance parameters for biocomposites of GOx@ZIF-zni obtained with 60 mM Zn^2+^.

Parameter/Ratio of Zn:Im	1:100	1:50	1:20	1:10
Y_A_ (%)	100 ± 0	100 ± 0	99.63 ± 0.13	96.72 ± 4.58
Y_P_ (%)	n.d.	n.d.	n.d.	25.70 ± 3.05
P_loading_ (mg/g_carrier_)	n.d.	n.d.	n.d.	18.28 ± 0.18
Specific activity bicomposite washed dH_2_O (U/g_biocomposite_)	0.05 ± 0.06	162.84 ± 26.02	1212.34 ± 275.06	1319.96 ± 197.34
Specific activity (U/mg_enzyme bound_)	n.d.	n.d.	n.d.	28.01 ± 0.48
η (%)	n.d.	n.d.	n.d.	31.96 ± 9.53
Specific activity bicomposite washed dH_2_O and 10% SDS (U/g_biocomposite_)	0.16 ± 0.05	135.68 ± 17.15	313.71 ± 68.71	792.35 ± 114.03

n.d., not determined due to interference from imidazole in protein concentration measurements after biomineralization.

## Data Availability

All data regarding the study will be available upon request.

## Abbreviations

The following abbreviations are used in this manuscript:

SDS	Sodium dodecyl sulfate
ZIF	Zeolite imidazole frameworks
HmIM	2-methyl imidazole
Im	Imidazole
GOx	Glucose oxidase
PXRD	Powder X-ray diffraction
SEM	Scanning electron microscopy
BET	Brunauer–Emmett–Teller
